# Humanities, criticality and transparency: global health histories and the foundations of inter-sectoral partnerships for the democratisation of knowledge

**DOI:** 10.1057/s41599-020-0491-7

**Published:** 2020-06-17

**Authors:** Sanjoy Bhattacharya, Alexander Medcalf, Aliko Ahmed

**Affiliations:** 1 Centre for Global Health Histories, Department of History, University of York, York, UK.; 2 Department of History, University of York, York, UK.; 3 Cambridge Institute of Public Health, University of Cambridge and Public Health England, Cambridge, UK.

## Abstract

Historians of medicine have been influential actors in a broader movement to highlight the social, institutional and administrative benefits of historical research, and its relevance for national and international policy intended to extend and improve contemporary healthcare. Historical perspectives are fundamentally useful to health policy actors because questions about what it is to be healthy, to suffer disease or disability, and the presentation and acceptance of solutions are interwoven in culturally and historically complex webs of meaning. Historians, as they have examined the social and cultural social determinants of health, have also used their work as public engagement, educational and policy resource tools, demonstrating that history is an effective way of making key issues in science, medicine and well-being more administratively responsive and accessible to lay audiences. This article explores such issues through the case study of the long-running World Health Organization (WHO) Global Health Histories project. Established in late 2004, the project’s enduring rationale has been that understanding the history of health helps the global public health community to respond to the challenges of today and help shape a healthier future. It has sought to do this by bringing together researchers and policy-makers into honest and democratic conversations and exchanges of ideas. The aim has been to stimulate a fusion between historical evidence and current policy approaches to many of the most urgent health issues. This article discusses the challenges and opportunities in bringing health history and policy together, and explores the importance of explaining historical method and the need to convince policy partners how history is evidence-based, that it can access and provide useful strategic information from archives of major institutions, and, therefore, a useful contributor to making policy initiatives adaptable and acceptable within complex polities and societies.

## Introduction

Historians of medicine have made significant contributions to the broader movement highlighting the relevance and social benefits of historical and humanities research to contemporary policymaking in a number of very distinctive contexts. In terms of health policy, as well as helping policymakers ‘extract real “lessons” from the past’ (Birn, 2009, p. 62), historical perspectives are considered particularly useful because questions about what it is to be healthy and to suffer disease or disability are interwoven in culturally, socially and politically complex webs of meaning. Historians probe these meanings critically, the complex negotiations that underpin them, and evaluate decisions concerning the impact of administrative contexts, moral calculations and formal ethical protocols, often working alongside colleagues from other disciplinary backgrounds to better integrate the humanities, social sciences, biomedical sciences, medicine and public health. Stevens, Rosenberg and Burns maintain that without history, thinking about health policy in our complex present would be an impossible task (Stevens et al., 2006). Pickersgill et al. agree, arguing that public health and global health are ‘unimaginable without the insights of qualitative and quantitative social sciences’ (Pickersgill et al., 2018, p. 1462). These examples are part of an increasingly global conversation about the fundamental benefits of historical research to policy.

This article discusses the opportunities and challenges in bringing health history and international health policy together through the case study of the long-running World Health Organization (WHO) Global Health Histories (GHH) project. GHH was founded within the WHO Headquarters in Geneva (WHO HQ) on the principle that academic history could contribute productively to present-day debates about health challenges, and whilst the project’s title has remained unchanged, for several years it has also invited and embraced a variety of perspectives from different disciplinary backgrounds, to look at the past by drawing on diverse kinds of sources and voices in a multiplicity of ways. Established in late 2004, mainly through the energies of Dr. Ariel Pablos-Mendez (who at the time was the Director of the former Department of Knowledge Management and Sharing, within the WHO HQ Division bearing the same name), the series’ enduring rationale has been that understanding the history of health helps the global public health community to respond to the challenges of today, thereby shaping a healthier future. GHH has sought to do this by bringing together researchers and policy-makers, predominantly through seminars, meetings and engagement events held at WHO offices and venues around the world, using the presentations and the resulting discussions to stimulate a fusion between historical evidence and current approaches. It has sought to help construct new and enduring bridges between academia and global health policy, while at the same time promoting public engagement and raising awareness around key issues in global health.

The benefits of increased dialogue, knowledge sharing, and collaboration between historians (and indeed scholars working across the humanities and beyond) and policymakers have been diversely acknowledged. However, the broad enthusiasm to strengthen and deepen these relationships comes with the need to acknowledge and work with a number of important caveats. The history in question needs to be rigorously practiced and independently prepared: historians must be allowed to uphold their criticality in order to capture the widest possible range of voices, opinions and experiences, and at the same time avoid the production of hagiographies or simplistic ideas of unchallenged and uncomplicated progress (generally based on narrow and over-generalised descriptions of policy contexts). Institutional histories are often triumphalist (even if, sometimes, not openly so), highlighting the work and contributions of a relatively small number of actors to the exclusion of others and the many complex administrative structures that were involved in all aspects of healthcare delivery including major international and global health programmes that only succeeded due to significant regional cooperation and national government contributions (Bhattacharya, 2006; Bhattacharya, 2013; Bhattacharya and Campani, 2020).

The constant search for independence and criticality are part of enduring scholarly anxieties about research potentially being misappropriated and misrepresented in subjective political and public health contexts to justify very specific, contemporary policy choices. Virginia Berridge cautions against the tendency towards ‘historian free data’, whereby history is used, but without the involvement of historians (Berridge, 2008, pp. 319–20). In a similar vein, Sally Sheard writes that the popularity of history has ‘enabled some UK health policymakers to feel they can “do it themselves” to the exclusion of professional historians’ (Sheard, 2018, p. 141). The report ‘What is the Value of History in Policymaking?’, commissioned by the UK’s Arts and Humanities Research Council (AHRC) and Institute for Government in 2015, concluded that ‘understanding what history is and how it can help is an important part of making good policy decisions’, but that a ‘more systematic approach to embedding history within the policy process’ was required (Haddon et al., 2015, p. 3). How to convince policy partners of the value and relevance of history, and create inter-sectoral partnerships to allow productive collaborations to flourish, have therefore also been at the forefront of debate for many years now (even if this work has dealt mainly with specific national contexts, rather than the much more intricate and expansive networks involved in planning and delivering international and global health projects).

At the same time, there needs to be a realistic and thoughtful appreciation of what history can bring to these discussions and how it can be used most effectively to challenge assumptions, stoke debate, conceptualise alternative approaches to present day challenges and enable a non-hierarchical (democratic, if you will) sharing of policy-relevant findings and data amongst wide-ranging, complex administrative constituencies. Even as ‘lessons’, in the form of thoughtful narratives about complex official and societal attitudes and actions from the past, are in some cases expected and desired by audiences outside of academia, it is erroneous to believe that historical research can be used to extract simple instructions about what worked or failed in the past, and use these as a schematic for planning the future. Although the potential benefits of closer working relationships between historians and policymakers have been widely advocated, getting research noticed by national and international administrators, and establishing the requisite collaborations to make this more likely are also rarely straightforward. Indeed, further questions about how these collaborations are then evidenced have also come to the fore, particularly since the requirement to demonstrate the impact of research has intensified. Academics working within the arts and humanities have felt increasingly pressured to demonstrate value and relevance (Hazelkorn, 2015), but as King and Rivett argue, historians can face a struggle to evidence the relative extent of their research, particularly when it falls outside mainstream definitions of impact, and therefore risks not being captured or valued (King and Rivett, 2015). Ultimately, whilst there is a strong record of historians engaging policy effectively and ethically, to a certain extent what forms the collaborations should take, and how the impact of these should be measured and quantified, are still being worked out.

The purpose of this article is to consider connected points of concern. Can academic historians really make a difference to the inter-connected regimes of national, regional, international and global health work today? How can they ensure that the voices of the few from one location are not simplistically presented as the universally accepted view or solution? How can historians help differentiate between what are very distinct and complex processes of policy design, implementation and evaluation, which take place at multiple sites involving actors from across the political and health spectrum? The present article also speaks to wider questions about historians’ attempts to bypass institutional myths about the top-down imposition of small sets of supposedly unified ideas and principles, and study large, multi-faceted organisations and their interconnections with other, similar bodies in the greatest possible complexity. The nuanced study of the genesis of plans and projects, and the implementation sciences underpinning them, play an enabling role in forming inter-sectoral partnerships for the democratisation of evidence collection and use, involving data collected from and in partnership with local governance and target populations.

In the ensuing discussion, this article will reflect on the challenges associated with convincing policy partners involved in the GHH project of the value of critically conceived historical methodologies, which push back against over-generalisation, simplification and exclusion in the collection of data about qualitative aspects of health planning and administration. Above all, it unpacks how such projects can contribute to the democratisation of evidence collection and knowledge production about health and medical policy. This builds on, and contributes to, the idea of ‘healthy publics’ as ‘dynamic collectives of people, ideas and environments that can enable health and well-being’. As in the case presented here, these publics may be geographically or spatially diverse, but bring to bear a range of expertise, materials, relationships and experiences to ‘question received or established approaches to health’ (Hinchliffe et al., 2018, p. 2). The present article thus demonstrates how those with expertise and experience in various fields can collaborate to enable health, and explores what it takes ‘to mobilise and sustain transdisciplinary groupings that can redefine and improve health and well-being’ (Hinchliffe et al. 2018, p. 4). It also shows how important it is to consistently listen to, record and report the widest possible range of voices, from all sections and levels of governance and society. As we shall argue in the sections that follow, our work with WHO partners rejected all efforts at creating subjective norms that are presented as being valuable for universal application; thoughtful humanities and social science work of value to creating democratic spaces in national, international and global health policies constantly iterates the importance of studying diverse contexts in their own terms.

It has also been demonstrated that history can be an effective way of making key issues in science, medicine and public health more accessible to different audiences, as well as helping to explore and understand complex issues. The methods and strategies for doing this have expanded significantly in the last two decades: whilst the opportunities afforded by the digital world, such as social media, should not be seen as an unproblematic route to ‘engagement’, the tools now widely available have arguably reshaped this practice. We agree with Hinchliffe et al. here as well, that engagement is not a one-dimensional approach to creating an informed citizen, but striving as far as possible for a more involved, collaborative approach (Hinchliffe et al., 2018). Innovative projects have been developed to help ensure that people feel a part of, and able to contribute towards the research process, and to help make project teams and outcomes more immediate and accessible. Rather than simple dissemination, interaction, engagement and institutional uptake are the main watchwords, and projects have made use of multi-pronged approaches, combining traditional elements like public lectures and exhibitions, with more ‘hands on’ approaches. GHH has ‘reached’ out extensively since it was founded, and we take this opportunity to also reflect on the potential, the potential pitfalls, and the consequences of the different strategies for doing so.

In preparing this article the views and opinions of twenty-two individuals connected to the GHH project from within and outside of the WHO have been invited (thirteen of whom provided responses). They were requested to be honest and critical about the opportunities presented by the series and the challenges that it has faced, and we are delighted to feature their voices as part of this reflection. We offered the opportunity for these views to be reproduced anonymously, but several have agreed for this anonymity to be waived when informed consent was obtained.^1^ In addition, in analysing them we have approached these reflections critically, and recognise that some are recalling decisions and events which took place more than a decade ago. They convey a sense of satisfaction at being involved in the planning and running of these events (which, again, is a lesson to try to make such projects fulfilling for all concerned), but also concrete reflection on the problems of maintaining influence, negotiating a multifaceted and ever-changing organisation, and demonstrating impact. The interviews are used to supplement published contemporary reflections, captured periodically in publications such as *Wellcome History.* Taken together, we use these sources to reflect on the project and offer what we feel historians might take away from this specific example of how to negotiate with and how to speak with non-historians. We hope that the ensuing discussion will provide useful information to those involved in, or seeking to establish similar projects, as well as those looking to engage policy on an individual level.

### The global health histories project

The GHH seminars have run continuously for close to fifteen years. Over that time the series has surpassed 138 events; involved hundreds of WHO staff, government officials and academics from around the world; and has engaged with thousands more via audiences in the room, online, and through project materials at related events including public exhibitions. It is not intended in the space below to give an entire operational account of the series. However, in the absence of a collected history of this initiative, we seek to discuss the project’s beginnings and how it developed. Particular attention will also be paid to significant innovations that not only shaped the format and delivery, but which also ultimately influenced and expanded its goals—namely evidence collection, forming partnerships to analyse and explain complexities, and democratising the co-production of knowledge for transparency and equity in health planning and delivery. This will establish important context to the coverage of policy and public engagement in the article’s remaining sections, but will also demonstrate the intricacies and negotiations which have shaped the project.

The GHH seminar series was established in late 2004 when members of WHO HQ’s senior management, especially Dr. Timothy Evans, then an Assistant Director General, became convinced that it would be useful to the organisation to develop historical insights into the public health projects of the recent past. Dr. Evans brought in Dr. Ariel Pablos-Méndez as the Director to spearhead the development of the idea within WHO HQ, who in turn brought in Thomson Prentice, the editor of the *World Health Reports,* and, from 2005 onwards Dr. Hooman Momen, who headed WHO Press, as the officials responsible for the series’ day-to-day management. The vision was to help build understanding regarding the changing times and forces shaping the WHO and the field of global health more generally. Dr. Pablos-Méndez also invited Elizabeth Fee, Theodore Brown and Marcos Cueto to write an academic history of the WHO (which has now been published by Cambridge University Press in its Global Health Histories book series (Cueto et al., 2019). Marcos, involved since the project’s start, recalls that WHO Director General Lee Jong-Wook was also very interested in the history component as part of efforts to mark WHO’s 60^th^ anniversary in 2008 (interview: Cueto, 2019). The GHH seminars were set up to continue and extend this initial work.

In its early stages these seminars, held at WHO HQ, were given only a small internal WHO budget and team, and relied on the voluntary efforts of small numbers of historians based in Europe and North America, some of whom agreed to serve as an external advisory panel. In 2005, Thomson Prentice approached the Wellcome Trust Centre for the History of Medicine at University College London (UCL), UK, for technical expertise and financial assistance, which was provided through Dr. Sanjoy Bhattacharya’s involvement, as well as a range of strategic funding from the Wellcome Trust (both directly and from within the Wellcome Trust Centre at UCL’s budgets). These resources helped open up WHO GHH to international networks and influences, shedding its relatively narrow, largely Euro-centric and US-centric focus. This fundamental shift in vision allowed the involvement of historians who had carried out detailed research on health policies and social responses to them across WHO regions and member states, making GHH much more international in its orientation.

The kind of history on offer at seminars was, out of necessity, presented in brief (the events lasting a little over an hour), but retained a consciously and carefully-defended critical edge, being based on the presentation of in-depth archival research and honest discussions about various policy drivers within the organisation. Within the GHH seminars, the historian presented their research after which time was reserved for comment and questions from the audience. An exception and major triumph in these early years was a seminar that featured Dr. Halfdan Mahler, ex-WHO Director General, as speaker. Mahler was very protective of his privacy and avoided interviews post-retirement; for this reason the event was hugely popular, attracting attendees from across UN agencies, universities and the international press. In an annual review, Thomson Prentice reported that Dr. Mahler’s contribution was enthusiastically received by the audience of over 150 current and former staff members, and provided ‘the first real evidence that the WHO has a deep and abiding interest in the history not just of its own work but of global health in general’ (Prentice, 2008, p. 9).

Indeed, the audience in these early stages was primarily composed of serving and retired WHO staff, and the public record of the seminars was minimal. Only the presentation slides were subsequently made available on a dedicated project webpage –though this reflected challenges associated with the resources and technologies available to the project, as well as the WHO HQ generally, rather than the aspirations for the series. The seminars nevertheless succeeded in the early aim of enhancing discussion and debate between academics and policy makers on a variety of pressing health issues (Prentice, 2009). Crucially however, they also demonstrated an appetite for critical historical insights amongst staff at different levels and departments within WHO HQ, and from regional offices and national government officials who attended these events.

The project shifted direction in 2009 when it was placed under the formal coordination of Dr. Momen, the Coordinator of WHO Press. WHO GHH was now based within a restructured department for Knowledge Management and Sharing (which was later renamed Knowledge, Evidence and Research (KER)). This period also coincided with the arrival of a new KER Director, Dr. Najeeb al-Shorbaji, who was enthusiastic about the goals of the project and set about further internationalising its scope. This built on a major WHO report on Knowledge Management, published before his arrival in Geneva from the WHO Regional Office for the Eastern Mediterranean in Cairo, which had set out a specific strategy to help the WHO become a better learning organisation. The strategic publication declared that health systems were increasingly complex, and that this intricacy of practice was driven by historical, political and economic change. As such, decision-makers, health professionals, communities, and WHO staff needed to be able to find, use, manage and share knowledge, but also, very crucially, have the competencies and tools to do so. Within the report the WHO GHH project, in all its forms, was cited as a core official function, benefitting knowledge management and sharing through analysis of significant public health developments, milestones, trends and perspectives, and developing expertise in extracting and applying the lessons learned in public health (WHO, 2005, pp. 1–17).

Using the opportunities for change that these administrative shifts allowed, the seminar format was restructured to include extended commentary from a WHO staff member following the academic presentation. Here, Dr. Momen and Dr. al-Shorbaji ensured that the programme of activities was all-embracing within the WHO, involving officials from all clusters and their departments, and collaborating more closely with the many WHO colleagues who understood and promoted inter-sectoral action on the basis of inter-disciplinary evidence collection and analysis. This was intended to increase dialogue and create opportunities for lasting interaction, emphasise the contemporary relevance of the seminars, and also enable WHO staff with an interest in understanding the complex pasts of present day challenges to create new discussions and pioneer new research methods across departments.

2009 represented the first time an entire annual GHH series tackled an overarching theme, namely tropical diseases, with the express goal of bringing in WHO HQ-based formations that did not always work together closely: the Neglected Tropical Diseases department, and the United Nations Special Programme for Tropical Disease Research (which had its offices within the WHO building in Geneva at the time). As part of the effort to significantly reduce the burden of tropical diseases such as Chagas disease, dracunculiasis, human African trypanosomiasis, leish-maniasis, leprosy, and onchocerciasis, a seminar was held on each of these health challenges enabling historians from across the world to participate in the effort by offering a different way of thinking about and approaching them (World Health Organization, 2009). The series involved a senior government official for the first time, in the form of Dr. C.P. Thakur, an ex-health minister and adviser to Indian agencies, in the seminar on kala azar. A well-known tropical diseases researcher, Dr. Thakur reported his findings from detailed studies on the ground in eastern India (World Health Organization, 2009). Another democratic landmark was reached in 2009, when a sustained effort was made to broadcast GHH events live and without any impediment of access over the internet.^2^ This meant that an even greater online audience could listen to the talks, view the presentations, and listen to and pose live questions in the discussion. Subsequent uploading of the recordings to the GHH website increased the longevity and potential impact of the events, serving as a readily accessible information resource.

In 2010, the GHH project’s academic coordination moved from University College London to the University of York, UK, with dedicated funding from the Wellcome Trust for continuation of work within the WHO HQ. In response, the initiative received increased support and legal recognition from the WHO’s management, becoming an official and audited part of the WHO programme of work for 2010–2011. It was now known, in WHO legalese, as an Office Specific Expected Result (OSER). This facilitated expansion of the work, involving all clusters and departments in WHO HQ. Wellcome Trust funding was renewed for the year and long-term support for a continuing programme of work was ensured through a Wellcome Trust Senior Investigator Award granted to Professor Sanjoy Bhattacharya in 2012. Recognition of the value and effectiveness of the GHH initiative was given the following year through the creation of a unique WHO Collaborating Centre for Global Health Histories based within the University of York,^3^ with GHH seminars, training workshops for WHO and member state officials, and other activities becoming part of an expanded official and audited WHO workplan.

Selecting the seminar topics was a collaborative effort: the WHO HQ-based coordinator sought topics that were timely and on which its departments would like to focus more attention. But the external collaborator, represented by the WHO Collaborating Centre from 2013 onwards, retained a say in the selection of academic speakers, ensuring that they were used in their professional capacity, and permitted to offer new insights and raise difficult questions. Although academic and WHO speakers were encouraged to interact in advance of the seminar, usually by meeting up and having frank conversations the evening before the event over supper, academic presentations were not vetted for criticism of the WHO. Thereby, all events celebrated the academic presenters’ independence and specific expertise; as everyone agreed, a democratic exercise that helped increase transparency of WHO structures to outside audiences helped all concerned. Similarly, following the official presentations the audience was invited to contribute to the discussion, and often the WHO’s viewpoint and performance was debated vigorously and freely, both by serving and retired WHO and UN officials, a fact which can be evidenced in many of the recordings which were subsequently made freely accessible online.

After 2012, the outlook of the seminar series was also fundamentally altered by the broadening of the voices involved and disciplines represented. Despite retaining the overarching title ‘Global Health Histories’, and a key focus on the historical contexts of topical issues in global health, seminars now involved scholars from a wide variety of disciplinary backgrounds. The seminars now incorporated a multiplicity of ways and methods for looking at the recent past, different modes of understanding voices and actions from the past, a wider range of data and its analysis, and, not least, a wider understanding of inter-disciplinarity. Bringing together different forms of research and analysis provided a more wholesome understanding of social and cultural determinants of health. This was frequently facilitated by increasing the number of panellists, which also reshaped the format of the seminars towards briefer commentaries and position papers, and allowed increased time for discussion and debate. This also facilitated longer, more detailed and newer conversations and research collaborations between academic participants, both before and after the event. Using social media platforms offered different ways for the project to reach out in addition to the live broadcasts.

Until 2013 the seminars were held exclusively in Geneva, but from that year onwards the project expanded to different WHO regional and country offices in response to demand from these contexts, further democratising the reach of the series within the agency’s complex worldwide networks. The first such event took place at the WHO Regional Office for Europe in January 2013, with a seminar dealing with what is now considered the urgent theme of anti-microbial resistance. The WHO GHH initiative’s managers were often approached by departments to help in the broadening of discussions about emerging problems, which, it was increasingly accepted, needed inter-disciplinary research that could drive forward multi-sectoral action (World Health Organization, 2013). May 2014 saw expansion to the WHO Regional Office for the Eastern Mediterranean with a seminar on the ‘Public Health Implications of Mass Gatherings’ (University of York, 2014). 2016 marked the first GHH seminar to be held with a WHO Country Office, in Sri Lanka, on ‘Universal Health Coverage and Sustainable Development Goals’ (University of York, 2016). This began a fruitful collaboration, with subsequent events receiving attention in Sri Lanka’s national media (The Island, 2017). Also that year, for the first time a GHH seminar was held in the Americas, at the Casa de Oswaldo Cruz, Fiocruz, Rio de Janeiro, Brazil (Fiocruz, 2016).

The 100^th^ GHH seminar in 2017, also held at Fiocruz, marked a further step in the internationalisation of the series, being live-streamed in English and Spanish to a global audience (Fiocruz/ GHH, 2017). This period was also marked by management shifts brought about initially by Dr. Momen’s retirement from the WHO in 2014. Day-to-day management of WHO GHH turned to Ms. Jing Wang-Cavallanti, a Technical Officer connected to WHO Press. Further, major administrative changes and reforms within the WHO followed, which impacted on GHH management and location. Dr. al-Shorbaji’s retirement led to the Department of Knowledge, Evidence and Research being broken up, with different sections being redistributed across other clusters inside the WHO HQ. At this point, in order to ensure its safety and retain its effectiveness, the GHH project moved its base, with active support from the Wellcome Trust, especially Dr. Simon Chaplin, to the WHO Regional Office for Europe’s Cultural Contexts of Health and Well-being (CCH) project, based in Dr. Claudia Stein’s Division of Information, Evidence, Research and Innovation, representative of the great interest for GHH seminars within WHO EURO. Indeed, Dr Stein commented that ‘I have been following them since their inception in 2004 and am really proud that we in EURO are now providing a home for them’ (University of York, 2016). This regional relocation of centre of operations boosted the internationalisation of the project; in partnership with WHO EURO, GHH started working with other regional offices, national governments and overseas universities on a regular basis, sparking an unprecedented interest and uptake of these events around the world. The University of York’s Collaborating Centre status was renewed in 2017 in recognition of continuing impact, with Dr. Zsuzsanna Jakab, then Regional Director of the WHO Regional Office for Europe (currently WHO Deputy Director General), acknowledging the valuable contributions made over the previous four-year period (University of York, 2017).

Further project funding and increased staffing during this time also meant increased opportunities to take the outputs of these seminars to publics around the world. Specifically this centred on the publication of multi-lingual, general histories of Tuberculosis (Medcalf et al., 2013), Tropical Diseases (Medcalf and Bhattacharya, 2014), Universal Health Coverage (Medcalf et al., 2015), Leprosy (Medcalf et al., 2016), and Mental Health (Kerrigan et al., 2017), which often combined perspectives covered in the seminars. These were produced as hard-copy and freely downloadable books, and although based on rigorous research featuring critical perspectives, were composed in such a way as to give a non-specialist audience the opportunity to engage with these subjects. Multi-lingual production was a further marker in the drive to reach out and democratise the project (in terms of international accessibility), and also support the WHO’s ePORTUGUÊSe network in making information available in this widely spoken, but not official UN language.^4^ The books featured images from various archives, which were then used to create related exhibitions shown around the world.

Over the years GHH has expanded the voices and disciplines involved in the discussions, the locations and audiences, as well as opportunities for knowledge exchange. The next section will explore further the outcomes of these changes, but will also demonstrate the attendant challenges that needed to be overcome in terms of reaching out to the audience, selecting topics, fostering collaboration, and overcoming logistical challenges to name but a few. In discussing the project with reference to reports, interviews, and our own experiences we also add to the enduring conversation about how those with expertise and experience in various fields can collaborate to enable health, and makes clear what it takes to mobilise and sustain transdisciplinary groupings (Hinchliffe et al., 2018, p. 4).

### Strategic insights and lessons

When the GHH project was initially established, the principal audience was WHO staff and policymakers, and the goal was to provide insights into the work of the WHO and its predecessors, as well as information that would benefit current staff regarding the history and identity of the agency. Key staff involved in the creation of WHO GHH did not need convincing of the value of history, and were the driving force, which undoubtedly helped the initiative gain traction. The seminars were intended to give WHO staff involved in proposing new policies an accessible way of understanding why current policies existed, as well as how policies had evolved and their impact on local populations. Dr. Momen felt that this was fundamental before new policies should be proposed and reorganisation implemented. The vision was not simply about ‘learning the lessons of the past’ or offering straightforward chronologies; GHH emphasised that whilst paying attention to past programmes was important, this was not simply a case of looking at what succeeded or failed and the reasons behind this. Seminars regularly emphasised the need to be cautious about previous successes and, as Dr. Momen states, presenters highlighted the many variables changing over time: what had worked well in the past may no longer prove as effective because of changing conditions (Interview: Momen, 2019). Therefore, as well as giving important information on the agency’s operational and organisational history, the additional value to the WHO was to emphasise vital aspects such as the enduring need for flexibility to wide-ranging economic, social and cultural factors, as well as the necessity for policy actors to invite and work with local input in countries and sub-national units where programmes were being implemented. WHO GHH research into the recent past helped identify diverse social and political networks that had helped engage communities during previous crises and campaigns, as well as helping to draw out detailed information about a great diversity of local innovations, the networks that had made underpinning information of such action possible, and, not least, the cultural and administrative mores that overseas workers needed to look out for. Everyone involved regarded this as an exercise in democratisation, where important voices from national contexts, and all sections of society and administration within them, were being recorded and being made available for all, including health planners, workers, funders and evaluators.

The seminars also provided a succinct and accessible way for WHO staff and interns to access fully-researched histories of the workings of this complex organisation. Project staff were frequently approached with queries about the best sources to provide an overview of the WHO’s history: clearly these people valued history as a way of comprehending the WHO’s mission and role, and understanding why certain structures and procedures were in place so as to better navigate them. GHH contributed to this need: Dr. al-Shorbaji recalls that, ‘for many new staff in WHO, it [GHH seminars] was an eye opener for them on global health issues that nobody has ever briefed them about it’ (Interview: al-Shorbaji, 2019). Alongside the historians, the presence of serving and retired WHO staff provided a different perspective where those who were directly involved could add their experience as ‘witnesses’. As Dr. al-Shorbaji commented, the retired WHO staff were ‘free from the political and employment pressures… After retirement the retired WHO staff has the freedom to express their personal views, of course without harming the integrity of the Organisation that (s)he worked for’. GHH seminars provided a forum for those involved in certain programmes to discuss and debate their experiences with historians working on these topics and serving WHO staff. This resource was not only deemed beneficial to WHO staff; a number of respondents recalled that colleagues from national missions and other organisations were invited to attend on occasions where logistics allowed, and on the whole appreciated the information and background that they gained as a result. Indeed, Dr Stein described the GHH seminars as ‘a true inspiration for all involved in international public health’ (University of York, 2016).

Interview respondents also acknowledged the value of seminars towards fostering an atmosphere of collaboration and mutual understanding, something we have found important in addressing the challenges which can be common when forming mixes of personnel, expertise, ideas and evidence (Hinchliffe et al., 2018, p. 5). This was assisted by the fact that seminars were convened over lunchtime, with staff bringing lunch with them or meeting beforehand for coffee to help create a relaxed air. The result of this could be seen in the promotion of inter-departmental conversations and exchanges. Seminars worked across the administrative structure of the WHO, covering topics relating to the work and interests of many technical departments. In Dr. al-Shorbaji’s words; ‘The health topic has always been multidisciplinary and multiple departments has stakes in it. This provided an opportunity for WHO staff from different departments to talk and listen to each other despite the organogram.’ The creation of such an atmosphere is a function that cannot easily be measured, but it is clear from the assessments of senior WHO officials who were charged with overseeing and auditing the impact of WHO GHH that the ability to foster a productive and collaborative atmosphere, as well as inter-departmental linkages including between UN organisations, was an important and appreciated outcome of the project.

Indeed, the GHH project was quickly identified as having the potential to spread information and foster collaboration between the WHO and other UN agencies. However, this was more challenging to facilitate, being frequently dependent on logistical factors. For instance, before live broadcasting of seminars was possible, inter-agency involvement was limited and largely reliant on direct invites. Others might have had a particular interest in the topic being discussed, have heard about it via a colleague or the speaker (the link already being in place), or were simply ‘in town’ at the right time. However, since the seminars have been housed at the WHO Regional Office for Europe in Copenhagen the project has benefited from swifter opportunities for connection afforded by UN city, a collection of offices specifically designed to promote inter-agency collaboration. The project has demonstrated that strategic vision needs to go in hand with an appreciation of logistical realities and how to work in and around these to best effect.

GHH has nevertheless sought to foster this for some years through, for instance, selecting seminar topics likely to have a broader interest. Recent experimentation with the seminar panel line-up (including representatives from a range of organisations) has helped to increase the cross-fertilisation of ideas and bring more agencies into contact with each other. Of his experiences of the seminar series, Mwai Makoka, Programme Executive for Health and Healing within the World Council of Churches (WCC), an organisation which is located nearby the WHO HQ, felt that his particular seminar had made a valuable contribution to help ‘enlighten our collaborators, especially WHO, that the WCC has been a valuable partner to them and that the partnership should not be abandoned even amidst socio-political changes’ (Interview: Makoka, 2019).

Of course, the GHH series has also carried benefits to the academics involved, according to those who have been interviewed in relation to previous seminar reports, and as part of this article’s research. There are several points of convergence: the ability to engage with an audience which they might not otherwise have had the chance to do so has been a strong feature of these reflections. This afforded historians experience in and the opportunity to hone their skills in communicating with non-historical audiences. A further aspect has been the audience’s ability to challenge speakers with a different level of background experience and expertise. Sally Sheard remarked that ‘It was good to present to an audience that was not purely academic or historical, as it encouraged me to rethink the context of the material I delivered’ (Prentice, 2009, p. 15), and Daniel Pick noted that he found the responses of the audience ‘interesting and helpful for my own continuing research on the topic I spoke about’ (Prentice, 2009, p. 15). Mark Jackson agreed with this, but felt that GHH had done more to ‘constructively develop two critical challenges’ facing historians of medicine: ‘firstly, the need to develop a more sophisticated global history that is not only sensitive to regional differences but is also transnational and comparative in its approach; and secondly, the drive to write a history of medicine that speaks more directly to scientists, clinicians, policymakers and the public’ (Prentice, 2009, p. 15).

The clear opportunity to increase contact with public health leaders and with officers of international organisations was also commended, but sustaining these is up to the individual, as well as a variety of external factors. The same can be said for opportunities to develop new collaborations and attend further events. For some speakers, of course, these would have been in place to begin with from previous work, but for others it provided a valuable opportunity to enhance critical skill sets and build connections. The new GHH series collaboration with Hughes Hall at University of Cambridge, which is developing an interdisciplinary ‘Bridge’ in global health that will bring together strengths from across college and university, is enabling such new connections and serves as a catalyst for inter-sectoral action where practitioners of history, the humanities and social sciences are encouraged to rethink their research, analysis and writing with a view to improving the outcomes of health programmes in the UK and the wider world.

The WHO GHH project has therefore benefitted from the support, energies and ideas provided by large numbers of organisational officials, who have welcomed the in-built criticality of the initiative, which allows independent researchers from outside WHO structures to present evidence and challenge stated positions within the organisation. However, such support was not always universal, especially during the period when the project was establishing itself, and this was often caused by time and resource constraints. Dr. Momen, Coordinator of WHO Press who provided day-to-day oversight within WHO HQ between 2005 and 2014, notes the challenges of setting up the initiative:

‘…all the work on GHH at WHO was carried out by staff members who were deployed in other functions and carried out the GHH work in addition to their other duties. There was also very limited financial resources, other than the staff time devoted, available from WHO. This was a severe limitation on being able to fully utilize the opportunities made available through the GHH project. Politically the project was fully supported at the Director, Assistant Director-General and Deputy Director General level. Overall there was enthusiasm about the seminars when the GHH was presented to the various department heads and co-ordinators. Some remained sceptical and needed more convincing…’ (Interview: Momen, 2019).

Other forms of resistance and push back were visible when the initiative was better entrenched within the organisation as an official WHO activity, caused mainly by discomfiture about the levels of openness and criticality, and often marked by conscious and unconscious biases about the speakers’ disciplines, background and, at times, origins. Here opposition to activities could take multiple forms. Refusal to attend was one action, but other approaches could also be seen: attendance of events in the final minutes, followed by uninformed aggressive questioning and, sometimes, the spreading of claims that seminars had not been well attended (even when events had struggled for space, with standing room only for attendees). Dr. al-Shorbaji enumerates the following challenges faced by the WHO GHH seminars:

1. Some of the middle management staff were not enthusiastic about the project and in some cases would create unnecessary barriers;
2. Selection of the topics had to be coordinated by different staff involved to make sure it’s timely, relevant and has potential for good audience;
3. Technological challenges in the form of broadcasting, recording, listening to remote participants;
4. The project was viewed at some point as a HQ activity and not related to regions and countries. This required doing some reaching out to get them on board. (Interview: al-Shorbaji, 2019)

As Sheard reminds us, we need to remember the need ‘to be realistic on the authority of historical knowledge in the policy arena’ (Sheard, 2018, p. 153). As an ‘extra’ activity, held over a lunch break, GHH required presenters and audiences to give up their own time, or time which, in a busy organisation, might have been reserved for other commitments. Not everyone’s interests could be catered to: selection of topics was a negotiation, with consideration given to what was timely and most relevant. In many cases the annual calendar of seminars was fixed in advance to facilitate travel and accommodation arrangements, but certain events were added at the last minute to facilitate coverage of pressing or emergency topics. These logistical challenges were often time-consuming; the kind of sticking points which will be familiar to most large-scale projects involving multiple stakeholders. The project has benefited from generous and sustained financial support, but has still depended on relatively limited resources in terms of staff time. From the WHO perspective, this has been particularly acute, and when staff were required for duty travel or asked to respond urgently during period of crisis, the seminars took a secondary role. This is, of course, understandable and a factor of working with such an agency.

As has been identified elsewhere, the GHH experience conforms to the recognition that convening diverse publics on health issues involves not only logistical but creative struggles over agenda, meanings and forms of working (Hinchliffe et al., 2018, p. 4). It is important to remember that the project was and is not simply about arranging and convening seminars on one particular day: each event hinges on significant in-person and teleconference meetings before and after events, at multiple locations, where wide-ranging, inter-disciplinary research groups have to be briefed, their support mobilised and their research made available in the form of accessible briefing documents. Such work is further underpinned by detailed preparatory research, in libraries and multiple archives, followed by the negotiation of focus of events, briefing of speakers and their managers, and, not least, enabling a meeting of the presenters the evening before so that presentational synergies could be robustly and democratically discussed. As Sheard discusses in ‘History Matters’ (Sheard, 2018), policymakers desire and expect answers/reports in days and weeks, rather than the months or years that historians usually work to, as the latter manage research and its analysis as a part of a much larger universe of academic activities that include student instruction and day-to-day pastoral care, public engagement and outreach, and all the associated administration. The challenge for the WHO Collaborating Centre team has been, however, to find ways to accommodate last-minute requests from within WHO frameworks for meetings around commitments within universities, which has sometimes strained arrangements in our home department. The priorities and calendars of WHO and government offices, and university departments working with them on WHO GHH seminars are frequently mismatched, requiring constant, dextrous and very difficult management.

Yet, the WHO’s senior managers responsible for overseeing, running and assessing the impact of the WHO GHH seminars clearly considered the initiative impactful. Indeed, gauging the precise nature of impact was something that project coordinators, within and outside the WHO, were always keenly aware of. This was to justify the use of resources and make the case for future funding, but also fundamentally to check progress towards the project’s objectives outlined above. Referring to the opportunities created by the initiative, Dr. al-Shorbaji identified a number of standout considerations:

1. Learning about the health topic from different perspectives;
2. Linking our staff with an outsider through knowledge sharing;
3. For many new staff in WHO, it was an eye opener for them on global health issues that nobody has ever briefed them about it;
4. For many policy makers and senior managers in WHO, there were some issues that they were not ready to open for discussion. This is either because WHO was not on top of the issue at the time or because they have current programmes that are dealing with the issue with little funding.

(Interview: al-Shorbaji, 2019)

A further consideration, the opportunity for WHO to highlight its programmes and policies, will be explored in section three below.

In essence, GHH created new synergies and spaces for interdisciplinary research and inter-sectoral policy collaboration, which were noted and acknowledged within the WHO leadership, which then enabled the release of staff time and resources for GHH to continue. The WHO GHH seminars were successful in creating bridges between different WHO departmental silos, which were being constantly reshaped even as its officials were being forced to work to punishing deadlines. They helped encourage wider thinking and cross-cutting work, which also enabled the drawing in of the expertise of retired WHO officials.

In this way, the WHO GHH seminars enabled wider connections with policy and created a learning and teaching resource. Each of these was formed under the overarching goal of helping disparate global public health communities better respond to present-day challenges and also to help shape a healthier future. Essentially, then, the objectives have centred on changing mindsets, institutional cultures and bringing in new forms of qualitative research and training in these methodologies into an organisation that has historically worked with relatively narrower quantitative evidence, mainly influenced by clinical and epidemiological understandings of disease and health management.

### Engaging with different publics: opportunities and challenges

As the GHH project developed, a balance needed to be sought between engaging and collaborating with policymakers, and reaching out to global non-academic, non-policy publics. Although a general international audience was not initially anticipated, project staff and supporters held that extending the reach to groups such as healthcare staff, journalists, and community groups, and others beyond, could help make a positive contribution to the democratisation of knowledge about the WHO, its programmes and practices, the history of international health, the present day global health milieu, as well as raising public awareness about important health topics. A recent seminar, on cycle helmet wearing, provided a powerful example of how members of the public can use the seminars to have a realtime discussion with WHO officials in ways they would be unable to otherwise, by putting questions to them and being able to engage in a live dialogue (Centre for Global Health Histories, 2019). This of course operates in both ways, with WHO officials and academic presenters able to interact directly with members of groups or the general public. With the advent of more reliable and accessible livestreaming technologies the vision to take the seminars to, and engage with, new audiences was progressively realised. This also fostered additional benefits in terms of bringing further transparency to proceedings, opening seminars up to wider critical comment, and creating an archive of free-to-access recordings and publications on a wide variety of pressing global health issues.

However, engaging on this scale was perhaps one of the most challenging and ever-changing aspects of the project. How to reach out effectively, make the programme inclusive, and evidence impact were all issues which required constant consideration. It was recognised that calculating the numbers ‘tuning in’ for instance was not an accurate marker of success. Of course, it is difficult to tell in what way each would have engaged with the ideas, and numbers alone did not sufficiently capture the diversity of engagement with the project. Regular ‘engagers’ knew, for instance, that a recording would be available shortly after the event, and if they did not have a specific question to ask, waited for the upload. Those in different timezones cannot, unfortunately, choose, and the question is how to give the ‘live’ opportunity to all. This is understandable; people are used to consuming media at a time convenient to them, yet it is important to retain a live-streaming function to uphold accessibility and transparency. However, such complexities needed to be factored in when evidencing impact, be it to the collaborating organisation, funder, or home department.

Capturing evidence of long-term engagement at relevant points raised different questions. Consistent attempts were made to ascertain how awareness of certain issues had changed as a result of the seminar, and how it might influence work or study. At many events, attendees reported that they had been encouraged to think differently about an issue, or were more aware of it than they had been before, but the fact remains, it is difficult to change mindsets through one seminar, or engage every segment of society. On this latter point, although this was unlikely to happen, project staff endeavoured to reach out on various media: communications were not limited in one direction, and the results of this were visible in the often surprising enquiries we received from around the globe. It has to be said however, that no matter how relevant, there are a realistic number of groups who will listen to the seminars. The effect must be cumulative over time, and make use of diverse methods of engagement (as discussed in more detail below).

A related question was also how to keep people connected with GHH over the long run, especially when they might be interested in one topic and not others. This was addressed partly through periodically rethinking how best to package and present the seminars to online and non-WHO audiences. In recent years the GHH format departed from the traditional seminar to a shorter event with punchier academic contributions and greater time for discussion. But, again, this was not simply about branding or ‘packaging’ the event: certain topics raised questions about how to sensitively treat and promote these to general and non-specialist audiences. Nevertheless, over the years the series has built up an effective ‘brand’, which is highly recognisable within WHO circles and externally as well. Alongside people following the GHH social media accounts, views of recordings continued to rise, and requests to be added to project mailing lists were received from around the world. However, this recognition brought its own issues. For one, it was judged that the name should stay the same, despite the fact that, as discussed above, the series became far more encompassing in terms of disciplinary perspectives. Furthermore, it became expected that there would be a historical component: various WHO departments sought exhibitions and engagement publications for many different topics, and were enthusiastic in their goals for this. But staff time and resources could not satisfy all requests. In this case we might also consider a disconnect between the historians and the WHO staff; there was an agreement on the value of historical research, but a distinct difference between the timescales that each worked to. To prepare project materials accurately and sensitively took a great deal of time.

The efforts to reach out and engage with wider audiences were extended and assisted by the aforementioned short histories, which built on the work of the seminars. These accessibly written, multi-lingual, open access books were distributed at related exhibitions and events in several countries around the world (including high-profile ones such as World Health Assemblies), and were uploaded as digital copies and made freely available online. In some cases these followed the format of the seminars: Tropical Diseases: Lessons From History (2014) was based on the 2009 GHH seminar series of the same name, and was intended to reach out and transfer to a wider audience the knowledge generated by these seminars. Each chapter was accompanied by a selection of images drawn from the WHO’s remarkable photo archive, as well as historic image archives such as the Wellcome Collection. Project staff found that the combination worked well in terms of grabbing the attention and encouraging people to read on, and the imagery assisted coverage in media outlets. Commonly received feedback was the request for more information on the images themselves, something addressed at the associated exhibitions, and was later turned into separate research projects with the outcomes again being fed back to the WHO.

The books have been reported as useful for a number of reasons within WHO: chiefly in terms of documenting, in a published format, the ideas, concerns, lessons, and obstacles facing healthcare professionals and policy makers, but also in terms of advocacy. Dr. Momen noted that the publications ‘provided a visible face for the GHH project and helped in presenting the project to other departments’ (Interview: Momen, 2019). Another respondent highlighted that publishing Leprosy: A Short History in Portuguese meant a great deal as Brazil, one of many countries covered in the book, has a high incidence and prevalence of the disease. The GHH series has a particular role to play in bringing to the public the strategies and actions of those involved in global health policies. History can help raise awareness about what these organisations are, what they do, how they work and bring them to public scrutiny, otherwise these realms of decision-making might appear important but otherwise inaccessible. Since becoming operational in 1948, the WHO has sought to develop ways to reach out and communicate with publics around the world about important matters in health (Medcalf, 2018), to keep the public informed about its work in a transparent manner, as well as to build a general interest in health matters. GHH has helped raise awareness of certain diseases, a factor which was appreciated by the individual WHO departments whose purview these were. By selecting topics of contemporary importance GHH seminars have afforded WHO a different opportunity to highlight the background and formation of its programmes and policies, and to explore, with a live audience made up of different perspectives, how and why these have been developed. The WHO has, however, historically been alive to the dangers of these efforts being seen as propaganda, and here again the critical nature of the seminars, and transparent nature of proceedings, ensured that reaching out to wider audiences was done equitably and openly.

More generally, there has been a degree of ‘re-use’, proving that the seminars and books can have a utility and value beyond the immediate present. The recording of Global Health Histories Seminar 110: ‘Polio, Immunization and Universal Health Coverage’ was played on a large screen for visitors at UN City Culture Night in October 2018. Featuring alongside medical historical artefacts including two iron lungs, this enabled a new audience interested in the question ‘what are vaccines and why do you need them?’ to access the material. Project books and exhibitions have also attracted the attention of non-academic outlets such as Huffington Post (Brooks, 2014) and Hyperallergic (Meier, 2014). It nevertheless remains difficult to gauge how mindsets have changed. Social media comments usually welcome the fact that the WHO is engaging with the particular issue in question, or say that WHO’s record in this area needs to improve. In these cases, the GHH seminars and associated activities succeed in stoking public interest in the agency, and bringing its work into closer scrutiny. Where possible more formal feedback has been sought by providing questionnaires after seminars.

There are a number of lessons, both for the project team but which we also feel are relevant more generally. In terms of reflecting on feedback and developing for the future, different ways of getting people’s voice heard have been explored such as collating questions that could not be answered on the day, and those generated through the recordings, and putting these to speakers. This extends the options available for people to question the WHO on its policies and actions, which provides a way of challenging dominant systems of knowledge, acknowledged as essential to the success of creating enduring healthy publics (Hinchliffe et al., 2018, p. 7). But this also requires sustained commitment on the part of speakers who are already giving up much time. In terms of reaching different audiences, the team at WHO EURO recognise that there is much more potential for expansion, and potential future ideas include hosting more screenings in country offices, possibly hosting a webinar in a country setting, and liaising more with country offices and national focal points on determining webinar topics of interest and finding speakers.

## Conclusion

The oral and archival history research carried out for all WHO Global Health Histories seminars and training events, as well as this article, shows how all academics keen to engage and partner national, international and global health programmes need toolkits to develop a full understanding of the great complexity of each of these contexts. The WHO, as it exists in Geneva, the Regional and Country Offices, and its partner governmental organisations at each of these levels are not monolithic organisations. WHO offices, and national and sub-national health ministries, are composed of multiple divisions and departments whose structure, staff and functions continue to change through processes of restructuring and reform. For academic bodies to consistently work on research and evidence-based policy management, it is, therefore, important that broad-ranging and robust alliances, built on mutual self-respect and trust are put into place. The WHO GHH project has been no exception. It has benefitted from developing lasting connections with multiple WHO Divisions, Clusters and Departments around the world, even though it has only been anchored within one Division at any specific point of time. This network of WHO alliances helped, in turn, to create wide-ranging links to national governments, which have been indispensable to the expansion and strengthening of the project’s many activities. This network of alliances is, perhaps, the principal determinant of WHO GHH’s longevity, representing the WHO’s longest-running, publicly-facing programme of work.

Whilst we have focussed on the WHO, a primary lesson from this article for future, similar activities is to develop robust understandings of—and relationships with—multiple departments and their personnel so that sudden reform, or departure or retirement of key collaborators does not diminish a collaborative programme or force it to close down. The development of deep, well-informed and mutually respectful relationships also promotes the democratic co-production and communication of research, enabling the articulation and recording, for future training and planning work, of the widest possible range of voices and viewpoints. Such an approach is reliant on the use of multiple languages, eschews the imposition of any social and cultural over-generalisations and Euro-centric norms, and is able to study polities and administrations in their own terms and consider context-specific understandings of problems and solutions. WHO GHH has consistently sought to underline the great significance of celebrating diversity and inclusion, and promote all forms of equalities and access during discussions about major challenges to health and well-being.

It is this ever-expanding, all embracing nature that has been one of the strengths of the WHO GHH project, and continues to define current activities and future plans. Working internationally with a range of leading universities, policy think tanks, government agencies, and learned colleges in medicine and public health, where all these organisations have contributed expertise and resources has been a boon. Increasingly, as these collaborations have developed, the York WHO Collaborating Centre’s role has been to help create links between WHO offices, member state governments, and leading regional and national universities, so that lasting cooperation can develop between them. The goal has been for them to work independently on delivering evidence-led considerations of the recent past and connections to present day challenges, and the development of thoughtful political and policy engagement on the basis of the knowledge created in collaboration. The ability of GHH to create new synergies, to democratise the production of (and access to) policy-relevant knowledge, to encourage new forms of inter-disciplinary research that connects academic and policy actors, and to optimise opportunities for inter-sectoral policy collaboration have been administratively useful in resource constrained contexts where external assistance in welcomed. Importantly, this is especially true when such engagement is inclusive and respectful of the existence of a multiplicity of viewpoints within each national, regional and international context, and the willingness to engage each in its own terms as collaborative, international efforts at health promotion is promoted within wide-ranging economic contexts. It is our hope that the information and reflections within this article will contribute to the enduring challenges of engaging and bringing together diverse perspectives and backgrounds to tackle pressing health issues. As identified by Hinchliffe et al., this conversation is ongoing and the strategies for forming the conditions for healthy publics will need to learn from and add to honest accounts of working together (Hinchliffe et al., 2018, p. 5).

## Data Availability

The datasets generated during and/or analysed during this study are not publicly available in order to maintain participant confidentiality and privacy.

## Notes

1 Informed consent was obtained from all participants. This research was approved by the University of York’s Arts and Humanities Ethics Committee.

2 This built on experiments in making audio available from 2008, with the seminars trialling the then new Webinar technology for the organisation.

3 WHO Collaborating Centres are ‘institutions such as research institutes, parts of universities or academies, which are designated by the Director-General to carry out activities in support of the Organization’s programmes’. For further background information see https://www.who.int/about/who-we-are/structure/collaborating-centres.

4 For further information on this network please see: https://www.who.int/eportuguese/mission/en/.
